# Primary cutaneous adnexal adenocarcinoma not otherwise specified versus metastatic breast carcinoma: A case report and review of the literature

**DOI:** 10.1016/j.jdcr.2024.05.041

**Published:** 2024-06-25

**Authors:** Nicole Babkowski, Gemma Savitz-Vogel, Angela M. Radoncipi, Jamie Stratton, Donald Savitz, Elgida R. Volpicelli

**Affiliations:** a Idaho College of Osteopathic Medicine, Meridian, Idaho; b Connecticut College, New London, Connecticut; c University of Connecticut, Storrs, Connecticut; d Department of Oncology, Stamford Hospital, Stamford, Connecticut; e Long Ridge Dermatology, Stamford, Connecticut; f Department of Pathology, Stamford Hospital, Stamford, Connecticut

**Keywords:** eccrine carcinoma, high-grade eccrine syringomatous carcinoma, immunohistochemistry, metastatic breast carcinoma, microcystic adnexal carcinoma, primary cutaneous adnexal adenocarcinoma, sweat duct carcinoma, sweat gland carcinoma

## Introduction

Adnexal adenocarcinoma is a skin tumor derived from apocrine or eccrine glands, hair follicles, and sebaceous glands. These neoplasms occur most frequently in the head and neck, and present as a single nodule or plaque of varying color.^1^ By histology, these tumors are often poorly circumscribed, infiltrative, and of heterogeneous composition consisting of tubules, nests, ducts, and glands.^1^ Rare characteristics include cellular infiltration in a single-file arrangement that resembles metastatic breast cancer (MBC).^2^ A wide variety of presentations in conjunction with nonspecific morphology and immunohistochemistry (IHC) can lead to diagnostic pitfalls and misdiagnosis, especially given overlap with visceral cutaneous metastasis. Primary cutaneous adnexal adenocarcinoma not otherwise specified (PCAANOS) is considered a diagnosis of exclusion, and cutaneous metastasis must be ruled out with histology, IHC, radiologic studies, and clinical presentation first and foremost.^1^

## Case report

**Fig 1:**
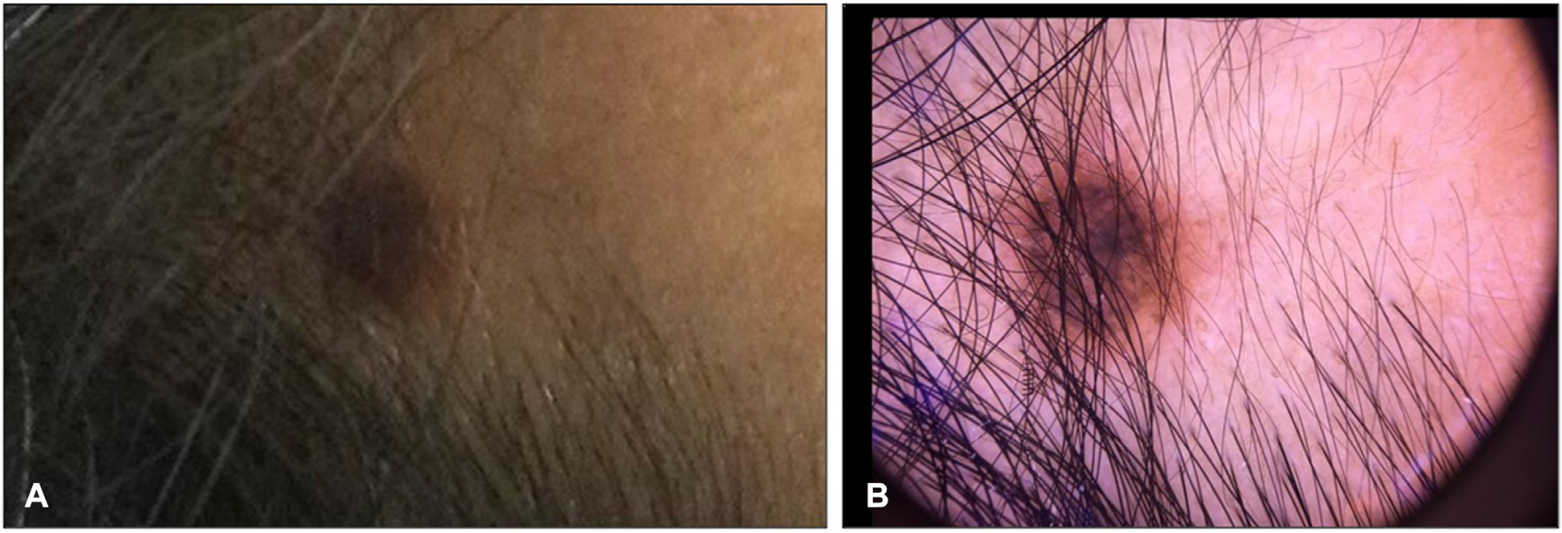
**A,** Clinical presentation demonstrating a solitary 9-mm hyperpigmented papule. **B,** Dermatoscopic close-up of hyperpigmented papule.

A 50-year-old woman with no significant medical history presented to her dermatologist for evaluation of a brown papule on the right hairline of 4-year duration. The patient reported slow change over the years with recent hyperpigmentation and increase in size. At the time of examination, a 9-mm hyperpigmented papule was present at the right frontal aspect of the scalp near the hairline (Fig 1, *A, B*).

**Fig 2:**
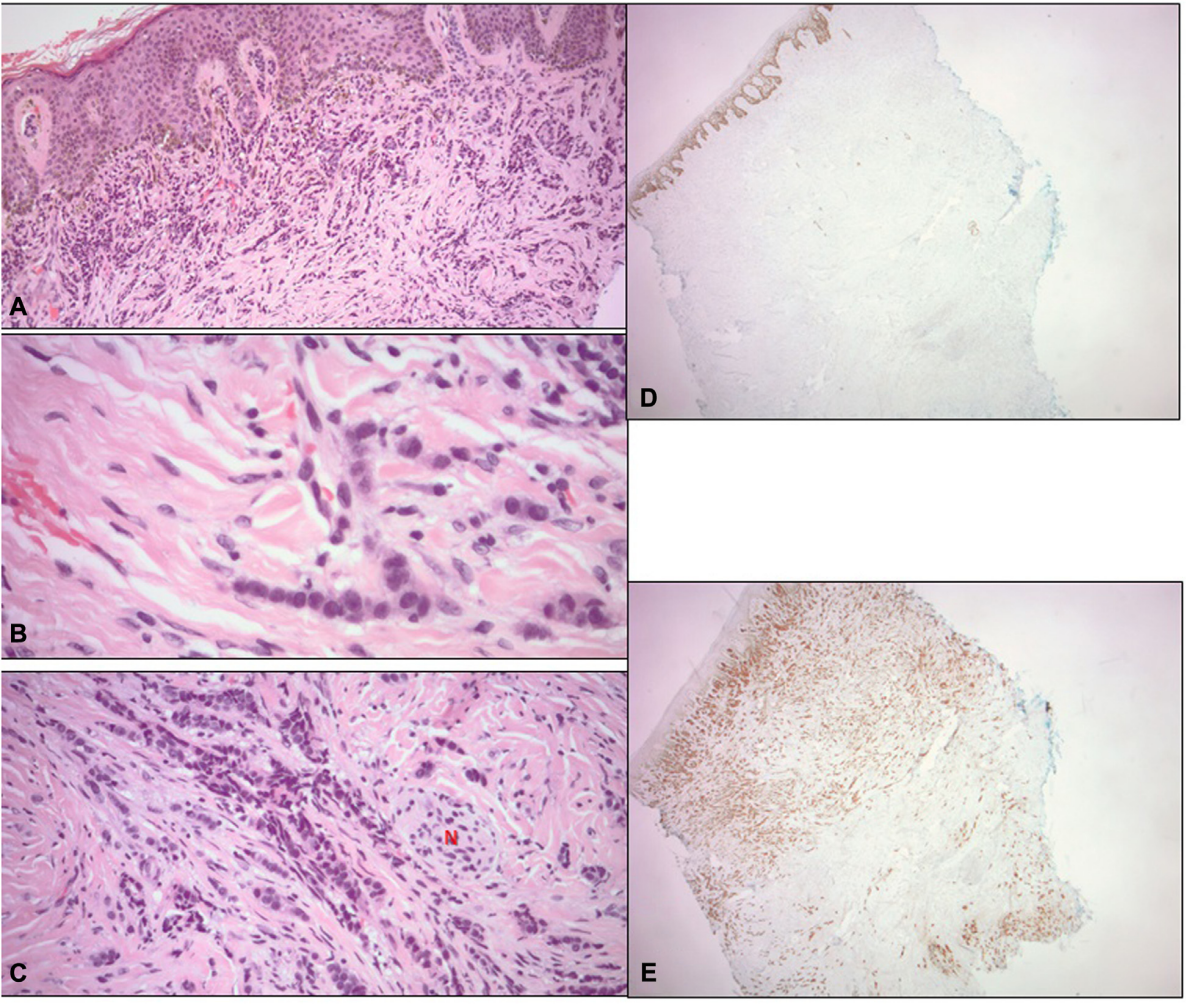
**A,** Busy dermis infiltrated by carcinoma cells. **B,** Single filing of tumor cells infiltrating dermis. **C,** The presence of perineural invasion. **D,** Complete absence of p63 staining within tumor cells. **E,** Strong and diffuse staining for mammaglobin by tumor cells. (Original magnifications: **A,** ×100; **B,** ×400; **C,** ×200; **D** and **E,** ×20.)

Patient denied pain or pruritus. The clinical differential diagnosis included a melanocytic nevus and dermatofibroma. A 6-mm punch biopsy was obtained. Pathologic examination identified a deeply infiltrating, malignant neoplasm composed of small epithelioid cells arranged in cords and nests permeating the dermis (Fig 2, *A*). The lack of striking pleomorphism and areas of tumor cells single filing in the reticular dermis (Fig 2, *B*) were morphologically suggestive of breast carcinoma, lobular variant, with the microscopic differential diagnosis including PCAANOS/sweat gland carcinoma.

**Fig 3:**
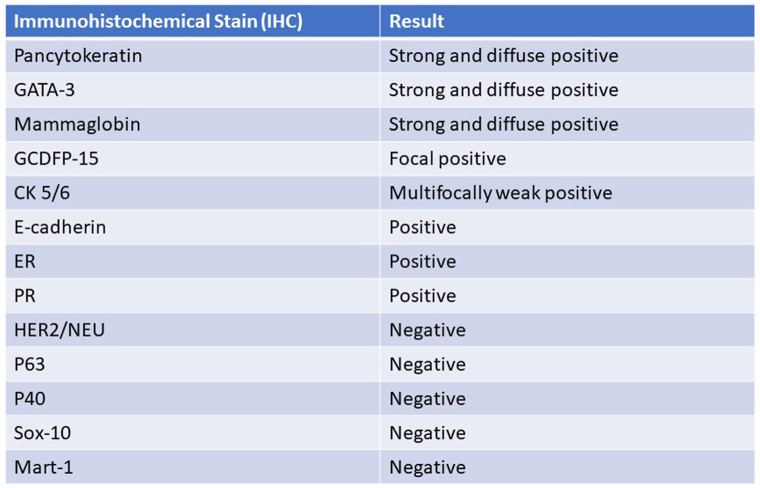
Table summarizing immunohistochemistry profile of tumor cells.

A wide panel of immunostains were performed the results of which are summarized in Fig 3.

Given the morphology and immunophenotype, the pathologic diagnosis was that of carcinoma infiltrating dermis with MBC being favored over PCAANOS and a disclaimer that clinical and radiologic correlation is necessary for definitive diagnosis. The slides were reviewed at 2 large academic centers with consensus opinion.

Subsequently, the patient underwent further work up which included a full body examination, a mammogram, breast ultrasound, and magnetic resonance imaging, all of which were unremarkable. A whole-body positron emission tomography scan was obtained, and it did not detect abnormally increased metabolic activity at any site to suggest a visceral malignancy or other sites of metastasis. A brain magnetic resonance imaging was also negative for metastatic disease. Constitutional review of systems and laboratory work up including carcinoembryonic antigen, cancer antigen (CA) 27-29, and CA 125 were all normal.

**Fig 4:**
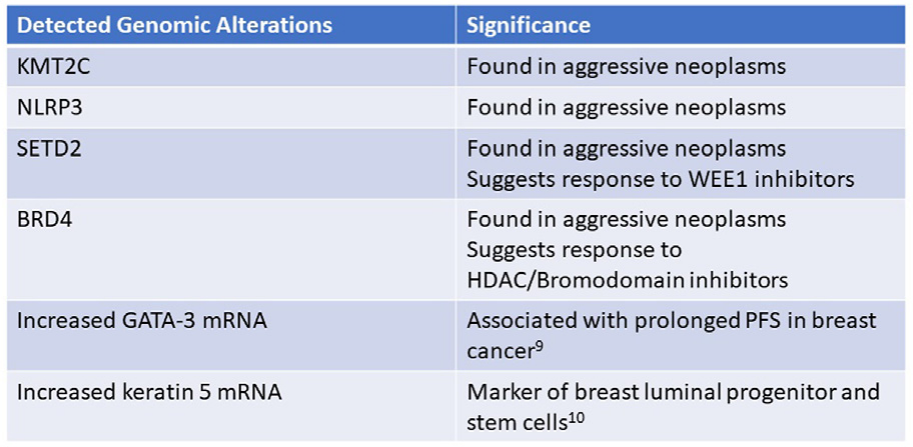
Table summarizing detected genomic alterations by solid tumor next generation sequencing and their clinical significance.^3^, ^4^ *PFS*, Progression free survival.

To further classify this tumor, the tissue block was sent for genomic profiling using next generation sequencing. The tumor showed no evidence of microsatellite instability and no evidence of BRCA 1/2, ESR1, or PIK3CA mutations. Tumor mutational burden was low and there was no homologous recombinant deficiency. There was no evidence of fusion messenger RNA involving ALK, RET, ROS1, or NTRK. Chromosomal structural analysis showed the tumor to have deletions of 7q, 10p, and 16q. Mutations of KMT2C, NLRP3, SETD2, and BRD4 were detected. There was also markedly increased GATA3 and keratin 5 messenger RNA (Fig 4). Overall, the findings were reported as suggestive of MBC with a note that the detected mutations are not likely related to UV radiation.

In the absence of clinical and radiologic primary visceral malignancy the patient was subsequently managed as having PCAANOS. The primary right frontal aspect of the scalp lesion was excised. Pathologic examination revealed residual tumor infiltrating dermis, subcutaneous tissue and superficial skeletal muscle with perineural invasion (Fig 2, *C*). Tumor extended to the deep margin and as a result a wider reexcision was subsequently undertaken.

Final pathologic evaluation showed a 1.9-mm focus of residual carcinoma involving the anterior/inferior margin. Given the positive margin post reconstructive surgery, the patient received radiation therapy to the right side of the scalp.

To date, the patient remains 5 months without recurrence, metastatic disease, or other significant events.

## Discussion

Albeit uncommon, cutaneous malignancies from visceral primaries are still more common than PCAANOS. In women, breast carcinoma is the most common culprit of cutaneous metastasis.^5^^,^^6^ As this case illustrates, MBC and PCAANOS cannot be reliably distinguished based on morphology and IHC alone.

**Fig 5:**
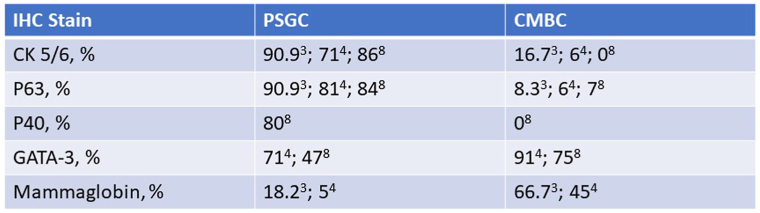
Table summarizing percentage of positive cases from 3 independent studies using a commonly used immunohistochemistry panel to distinguish between primary cutaneous salivary gland carcinoma (PSGC) and cutaneous metastatic breast carcinoma (CMBC).

Review of the literature highlights an attempt to use immunohistochemical panels to further aid in differentiating between MBC and primary sweat gland carcinoma (Fig 5).

GATA-3 staining has been correlated with both PCAANOS/sweat gland carcinoma and cutaneous metastasis of breast carcinoma, with one study finding nuclear positivity in 71% and 91%, respectively. As such, GATA-3 has no utility in distinguishing MBC from adnexal adenocarcinoma.^6^

GCDFP-15 was found to be infrequently positive in cutaneous adnexal tumors.^6^^,^^7^ This tumor, however, was GCDP-15 positive.

P63 and p40 have been promising markers as illustrated by various studies.^5^^,^^6^^,^^8^, ^9^, ^10^ Specifically, there was strong expression of p63 in 90.9% of the primary cutaneous adnexal carcinomas, and weak expression in 8.3% of metastatic breast carcinomas.^5^ P63 was also positive in all 14 reported cases of solid variant of microcystic adnexal carcinoma.^9^ P40 on the other hand appears to be the most specific marker for PCAANOS (92%) while retaining high sensitivity (80%).^10^ This tumor, however, was negative for both p63 and p40 (Figs 2, *D* and 3).

Mammaglobin was reported as positive in 66.7% of breast carcinomas and only 18.2% of cutaneous adnexal carcinomas.^5^ This tumor, was strongly and diffusely positive for mammaglobin (Figs 2, *E* and 3).

Overall, the immunohistochemical profile of this case with complete absence of p63 and p40 and strong and diffuse positivity for mammaglobin (Figs 2, *D*, *E* and 3) would argue for a diagnosis of MBC versus PCAANOS.

Ultimately, being able to reliably distinguish between these 2 entities is not of trivial pursuit because it means the difference between wide excision plus or minus radiation in the setting of PCAANOS versus chasing an occult primary and endocrine therapy in the setting of MBC.

As this case clearly illustrates the currently used IHC panels and even molecular studies lack to be desired. The gold standard remains clinical, radiologic, and pathologic correlation!

## Conflicts of interest

None disclosed.
